# Altered Metabolism of Blood Manganese Is Associated with Low Levels of Hemoglobin in Patients with Chronic Kidney Disease

**DOI:** 10.3390/nu9111177

**Published:** 2017-10-27

**Authors:** Minyoung Kim, Eun Sil Koh, Sungjin Chung, Yoon Sik Chang, Seok Joon Shin

**Affiliations:** 1 Department of Internal Medicine, College of Medicine, The Catholic University of Korea, 222 Banpo-daero, Seoul 06591, Republic of Korea; gomy33@catholic.ac.kr (M.K.); fiji79@catholic.ac.kr (E.S.K.); chungs@catholic.ac.kr (S.C.); ysc543@catholic.ac.kr (Y.S.C.); 2 Division of Nephrology, Yeouido St. Mary’s Hospital, The Catholic University of Korea, 10, 63-ro, Yeongdeungpo-gu, Seoul 07345, Republic of Korea; 3 Division of Nephrology and Hypertension, Department of Medicine, School of Medicine, Vanderbilt University, S3206 Medical Center North, Nashville, TN 37232-2372, USA; 4 Division of Nephrology, Incheon St. Mary’s Hospital, The Catholic University of Korea, 56, Dongsu-ro, Bupyeong-gu, Incheon 21431, Republic of Korea

**Keywords:** manganese, anemia, chronic kidney disease

## Abstract

Blood manganese (Mn) level has been reported to be higher in patients with anemia or iron deficiency. The purpose of this study was to analyze the relationship between blood Mn level and anemia in patients with chronic kidney disease (CKD). A total of 334 patients with CKD who were not treated with dialysis were included in this study. Blood Mn level and serum markers regarding anemia, renal function, and nutrition were measured and analyzed. Median blood Mn level was 8.30 (interquartile range(IQR): 5.27–11.63) µg/L. Univariate linear regression showed that blood Mn level was correlated with age (β = −0.049, *p* < 0.001), smoking (β = −1.588, *p* = 0.009), hypertension (β = −1.470, *p* = 0.006), serum total iron-binding capacity (TIBC) (β = 0.025, *p* < 0.001), serum transferrin (β = 0.029, *p* < 0.001), and estimated glomerular filtration rate (eGFR; β = 0.036, *p* < 0.001). Results of multiple linear regression analysis showed that beta coefficient of hemoglobin was 0.847 (*p* < 0.001) for blood Mn level in all participants after controlling for covariates, including gender, age, body mass index, smoking, diabetes, hypertension, and eGFR. Multivariate Poisson regression analysis with robust variance after adjusting for gender, age, smoking, hypertension, diabetes, eGFR, and nutritional markers showed that higher blood Mn level (per 1 µg/L increase) was associated with decreased prevalence of anemia (PR 0.974, 95% CI: 0.957 to 0.992, *p* = 0.005). Taken together, our results demonstrate that blood Mn level is positively associated with hemoglobin level in CKD patients. This might provide important information in the understanding of the pathogenesis of CKD-related anemia.

## 1. Introduction

Manganese (Mn) is an essential mineral that plays a role in the maintenance of human homeostasis. Several studies have reported its role in immune function, blood sugar, and cellular energy regulation as well as protection against free radicals [1,2]. Mn intake is mainly achieved through daily food intake. Green vegetables, teas, fruits, legume, grains, and rice are rich in it [3]. A sufficient Mn intake is usually obtained through diet. However, in patients with renal disease, especially those who are treated with dialysis, the amount of food and the content of the meal have limitations. Given that malnutrition is a major issue in chronic kidney disease (CKD) [4], Mn intake could be inadequate to maintain physiologic balance in CKD patients.

CKD is a chronic inflammatory disorder characterized by progressive deterioration of renal function that can be caused by oxidative stress. Inflammatory cytokines and uremic toxins not only affect the metabolism of protein, carbohydrate, and lipid, but also induce hormonal derangement [4]. Besides, these changes in CKD might affect the metabolism of trace elements. Alterations in trace elements including Mn have been observed in CKD [5,6]. How alteration of Mn affects the change of metabolism in renal insufficiency is currently unclear. Koh et al. [7] have reported that low Mn might contribute to excess oxidative stress in patients with uremia. Low Mn value in blood might favor renal disease progression [7]. On the other hand, a prospective study has shown a strong negative correlation between plasma Mn and estimated glomerular filtration rate (eGFR) [8]. 

Several studies have reported that blood Mn level is higher in patients with anemia or iron deficiency [5,9,10]. Mn and iron are structurally similar. They share many protein transporters such as divalent metal transporter-1 (DMT-1), by which they are competitively absorbed into the mucosal wall [9]. Although anemia is one of the most common manifestations of CKD, whether blood Mn level has some effects on anemia in CKD patients is currently unclear. Therefore, the objective of this study was to analyze the relationship between blood Mn level and anemia in patients with CKD.

## 2. Materials and Methods

From March 2014 to January 2016, 426 patients with CKD were enrolled from The Catholic University of Korea Yeouido St. Mary’s hospital, Seoul, Republic of Korea. Patients with renal replacement therapy, missing values, acute illness including pneumonia, urinary tract infection, bleeding and sepsis, or an age of less than 20 years were excluded. Medical records of patients were reviewed retrospectively to obtain data of demographics, clinical findings, and additional treatments such as renal replacement therapy. This study was performed according to the Declaration of Helsinki and approved by the Institutional Review Board of The Catholic University of Korea Yeouido St. Mary’s Hospital (approval number: SC17RISI0009).

### 2.1. Definitions

CKD was defined when eGFR was less than 60 mL/min/1.73 m^2^. eGFR was calculated by the Modification of Diet in Renal Disease (MDRD) study equation: eGFR = 175 × (standardized SCr)^−1.154^ × (age)^−0.203^ × 0.74 (if Asian) × 0.742 (if female)] [11]. Anemia was defined as blood hemoglobin (Hb) concentration < 130 g/L (<13 g/dL) or hematocrit (Hct) < 39% in adult males or Hb < 120 g/L (<12 g/dL) or Hct < 37% in adult females [12]. Transferrin saturation (TSAT) was calculated as serum iron level divided by serum transferrin.

### 2.2. Laboratory Measurements

Blood Mn level was measured using an atomic absorption spectroscopy method with SpetrAA-220 (Varian medical systems, Inc., Palo alto, CA, USA). The reported upper limit of blood Mn level was 8 µg/L. Serum ferritin was examined with electro-chemiluminesecence immunoassay (ECLIA) using E-170 (F. Hoffmann-La Roche Ltd., Basel, Switzerland). Other laboratory data including serum Hb, Hct, red blood cell distribution width (RDW), serum iron, serum ferritin, and transferrin iron binding capacity (TIBC) were also reviewed. Samples were acquired from the single blood draw at the time of admission and not serially checked.

### 2.3. Statistical Analyses

Continuous variables with normal distribution are expressed as means ± standard deviation. Data with skewed distribution are presented as medians (25–75% interquartile range). Categorical variables are described as frequencies or percentages. Spearman regression analysis was used to analyze the correlation between blood Mn level and other variables. Poisson regression models with robust variance were used to explore associations of blood Mn level, other covariates, and anemia. All statistical analyses were performed using SAS version 9.3 (SAS Institute, Cary, NC, USA). Statistical significance was accepted at *p* < 0.05.

## 3. Results

### 3.1. Basal Characteristics and Distribution of Blood Mn Level

**Figure 1:**
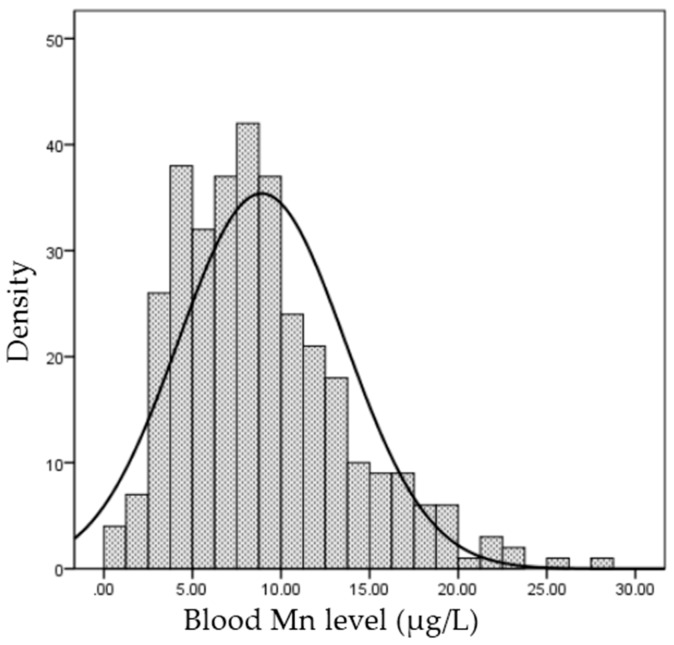
Distribution of blood Manganese (Mn).

A total of 334 pre-dialysis patients were analyzed in this study. Median blood Mn level was 8.30 (IQR: 5.27–11.63) μg/L. Blood Mn levels showed a non-normal distribution (Figure 1). 

Basal characteristics of these subjects are summarized in Table 1.

**Figure 2:**
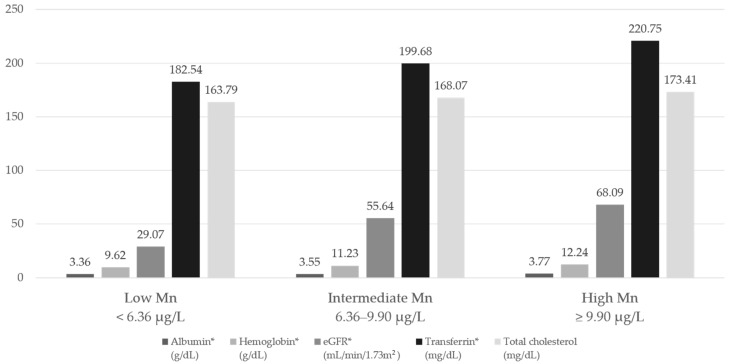
Nutritional markers according to blood Mn groups, * *p* < 0.05. Abbreviations: eGFR: estimated glomerular filtration rate

When we divided subjects into tertile groups according to the blood Mn level, low Mn group showed lower Hb level, and proportion of anemia was high (percentage of anemia patients by Mn groups: 90.0%, 68.8%, and 51.3%, respectively, *p* < 0.001) as expected. Levels of other nutritional markers including transferrin, and albumin were also low, but total cholesterol did not show statistical significance (Figure 2).

### 3.2. Determinants of Blood Mn Level

Univariate linear regression showed that blood Mn level was correlated with age (β = −0.049, *p* < 0.001), smoking (β = −1.588, *p* = 0.009), hypertension (β = −1.470, *p* = 0.006), serum TIBC (β = 0.025, *p* < 0.001), serum transferrin (β = 0.029, *p* < 0.001), and eGFR (β = 0.036, *p* < 0.001). Multiple regression analysis after controlling for covariates showed that beta coefficient of Hb for blood Mn level was 0.847 (*p* < 0.001) in all participants (Table 2). On the other hand, blood Mn level was negatively correlated with diabetes mellitus (DM) (β = −1.453, *p* = 0.012) and serum albumin (β = −0.041, *p* = 0.935). Smoking for blood Mn had a positive beta coefficient (β = 2.069, *p* = 0.001). 

### 3.3. Linear Association of Blood Mn Level and Anemia Markers

**Figure 3:**
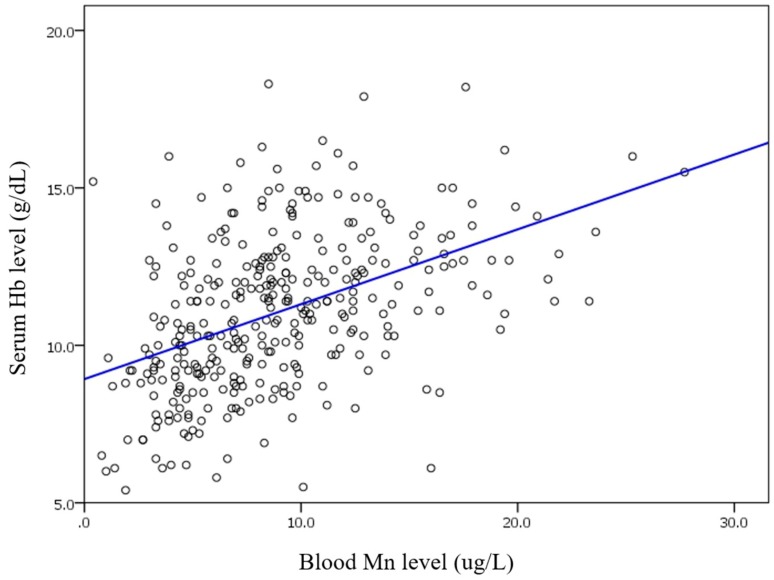
Blood Mn and serum hemoglobin (Hb).

**Figure 4:**
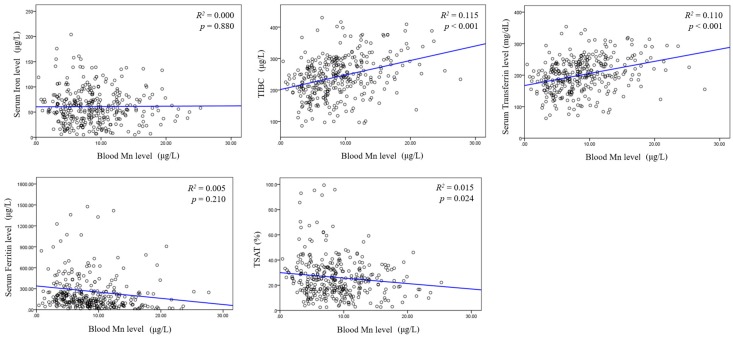
Blood Mn and various anemia markers.

In correlation analysis, blood Mn level was positively correlated with serum Hb level (*R*^2^ = 0.209, *p* < 0.001, Figure 3). Blood Mn level did not show any linear association with serum iron or serum ferritin level. However, linear association was observed between blood Mn level and TIBC (*R*^2^ = 0.115, *p* < 0.001, Figure 4).

To determine the association between blood Mn level and anemia markers, linear regression analysis was performed. In univariate analysis, TIBC (β = 0.025, *p* < 0.001), transferrin (β = 0.029, *p* < 0.001), TSAT (β = −0.035, *p* < 0.024), the use of erythropoietin stimulating agent (ESA) (β = −2.565, *p* = 0.001), and iron supplement (β = −2.099, *p* < 0.015) showed significant associations with blood Mn level. On the other hand, multivariate model revealed that TIBC was independently associated with blood Mn level (Table 2).

### 3.4. Poisson Regression Analysis with Robust Variance between Anemia and Blood Mn and Clinical and Nutritional Factors

To assess the association between blood Mn level and the prevalence of anemia, prevalence ratio (PR), and 95% confidence intervals (CI) were evaluated by Poisson regression model with robust variance. Multivariate Poisson regression analysis for anemia was performed after adjustment for gender, age, smoking, hypertension, DM, use of ESA, ferritin, transferrin, albumin, total cholesterol, and eGFR. Higher blood Mn level (per 1 µg/L increase) showed a decreased prevalence of anemia (PR 0.974, 95% CI: 0.957 to 0.992, *p* = 0.005). As expected, higher eGFR showed decreased prevalence of anemia (PR: 0.994, 95% CI: 0.991–0.996, *p* < 0.001). However, having DM increased the risk of anemia (Table 3). Nutritional markers including albumin and total cholesterol were related with a lower prevalence of anemia, which is similar to that of Mn. Post-hoc power analysis was performed, and the power was 0.999 (*f*^2^ = 0.15, α = 0.05, sample size = 344).

### 3.5. Prevalence Ratio (PR) and 95% Confidence Interval (CI) Values for Anemia with Blood Mn (μg/L) in Subgroups

To clarify the effect of blood Mn level on anemia, multivariate Poisson regression analysis with robust variance was performed after dividing all patients into two groups based on eGFR of 60 mL/min/1.73 m^2^*.* As shown in Table 4, blood Mn level only had a significantly negative association with anemia in the renal dysfunction group this association remained significant after adjusting for clinical factors, iron store, the use of ESA, and nutritional markers.

On the other hand, in subgroup analysis, according to the presence of DM, blood Mn level had a negative association with the prevalence of anemia in all models irrespective of DM, except for the subgroup with DM in Model 4 as shown in Table 5.

## 4. Discussion

Results of the present study show that blood Mn is positively correlated with serum Hb level. After adjusting for confounding factors, higher blood Mn level was found to be associated with a decreased risk of anemia independently in CKD patients. This is the first study where the association between blood Mn level and anemia was observed in CKD patients.

Previous studies have focused on the relationship between iron deficiency and higher blood Mn concentration [13,14,15,16,17,18,19]. Almost 50 years ago, Mena et al. [13] reported that 13 anemic patients aged 13–44 showed a 6-fold increase in red cell Mn concentration. Since then, a few studies have demonstrated that iron deficiency increases blood Mn concentration in adults, children, and infants [14,15,16,17,18,19]. Difference in blood Mn concentration according to anemia status has also been analyzed in a few previous studies [15,20]. Kim et al. [15] have shown higher blood Mn concentrations in anemic and borderline groups than those in normal participants. They presumed that such difference in blood Mn by anemia was due to ferritin status and total body iron store. Results of this study suggest that low serum ferritin concentration is a predictive marker of high Mn concentration, consistent with results of a previous study [15]. 

In the present study, median blood Mn concentration was 8.30 μg/L (Table 1), which was lower than that in the Korean general population with normal renal function [7,15]. However, this value is compatible with that of trace elements in CKD patients on hemodialysis [6,21]. An observational study has suggested that blood Mn deficiency might be involved in the pathophysiological process of renal dysfunction [7]. 

In baseline characteristics (Table 1), median values of iron profiles indicated that the participants were not deficient in iron store [22], especially for serum ferritin. However, serum ferritin is not only a marker of iron store but also an acute-phase reactant. It may be increased out of proportion in response to the amount of iron store in acute or chronic inflammatory disorders such as CKD [23]. Functional iron deficiency characterized by low serum transferrin saturation with normal or high serum ferritin is easily observed in CKD patients [24]. Functional iron deficiency is impaired when iron release from body stores is unable to meet the demand for erythropoiesis. This is called reticuloendothelial cell iron blockade [24]. Common established causes of anemia in CKD include relative erythropoietin deficiency, disordered iron homeostasis, circulating uremic-induced inhibitors of erythropoiesis, shortened red blood cell survival, and various metabolic and mechanical factors [24].

Recently, hepcidin excess has been found to account for impaired dietary iron absorption and reticuloendothelial cell iron blockade in CKD patients [24,25]. Hepcidin is a 25-amino acid hormone synthesized in the liver and secreted into circulation [26]. Hepcidin induces degradation of ferroportin, an iron-extrusion protein on duodenal enterocyte or reticuloendothelial macrophages, leading to the inhibition of iron entry into the plasma. [27,28,29]. Hepcidin translation is regulated by plasma iron level or concentration of the iron–transferrin complex [30]. However, inflammatory cytokines can also directly lead to hepcidin transcription in chronic disease such as CKD [31]. This is presumed as a defensive mechanism against invading pathogens, resulting in iron sequestration, hypoferremia, and anemia, the hallmarks of many chronic diseases including CKD [31]. One recent study has suggested that ferroportin and hepcidin play roles in iron and Mn transport [9]. Another study has reported that a mutation in gene required for normal synthesis of hepcidin might induce alteration of hepcidin, resulting in dysregulation of mitochondrial iron and Mn level [32]. Iron accumulation was observed mainly in the cytosol, but not in the mitochondria [32]. However, another study has reported that Mn level in the mitochondria is decreased, and such a decrease is associated with decreased activity of manganese-dependent superoxide dismutase (MnSOD) [32]. These results demonstrate the important interrelationship between iron and Mn homeostasis regarding hepcidin. They also imply a possible relationship between Mn level and hepcidin processing following the occurrence of anemia in CKD patients.

Malnutrition is very concerned in CKD in the aspect of interrelationship with inflammation, called malnutrition-inflammation complex syndrome (MICS), because malnutrition and inflammation are the main cause of the clinical outcome in CKD [33]. In early to moderate CKD, over 25% of patients have malnutrition and chronic inflammation, and as CKD progresses, more patients suffer from malnutrition and chronic inflammation [34]. In CKD, malnutrition is associated with not only diet but also hypercatabolism, inflammation, acidemia, uremic toxins, or oxidative stress [35]. In the present study, nutritional markers including serum albumin, transferrin, and total cholesterol were lower in the moderate CKD group than in the early CKD group, as expected (not shown). To clarify the effect of blood Mn itself on anemia, we conducted Poisson regression analysis with robust variance after adjusting those markers. In subgroup analysis based on renal function (Table 4), blood Mn showed a significant association with anemia only in the renal dysfunction group, even after adjusting for those nutritional markers. This independent effect of blood Mn on anemia in the moderate CKD group may be affected by not only malnutrition but also chronic inflammation and uremic and oxidative stress. Furthermore, Mn is a cofactor of the MnSOD enzyme, which contributes to defense against oxidative damage [36]. A previous study has demonstrated that Mn deficiency can lead to decreased MnSOD activity [37].

This study has a few limitations. First, this study had a retrospective design. Although a possible protective effect of sufficient Mn in anemia was noted, the actual role of Mn remained unclear. Randomized clinical study should be done to propose a target level for blood Mn. Second, although we performed multivariable models after adjusting for many factors, this study was performed with a relatively small sample size. Therefore, our results need to be interpreted cautiously because an unknown bias could not be entirely excluded. Third, blood Mn was checked from single draw of blood sample, not from the serial data which could be a temporary status. However, we have excluded subjects with acute inflammation to minimize this bias. Finally, we could not analyze the effect of medications, alternative medications, and eating habits on blood Mn because of a lack of information, which is a potential bias of our study. Despite these drawbacks, to the best of our knowledge, this is the first study that assesses the effect of blood Mn level on anemia, especially for CKD patients. Anemia is one of the most bewildering problems affecting the long-term outcome of CKD patients. Therefore, determining the optimal level of blood Mn in CKD might contribute to the management of anemia. 

## 5. Conclusions

We found that high blood Mn level appears to have an association with high serum Hb level in CKD patients. Malnutrition was also associated with anemia in CKD patients as expected. Given that anemia is a major concern in CKD patients, more attention should be paid to blood Mn for management of anemia in CKD patients. Further studies are needed to clarify the exact role of Mn in CKD pathogenesis.

## Figures and Tables

**Table 1:** Basal characteristics of 334 pre-dialysis CKD patients.

Characteristic	Total (*n* = 334)
Male, *n* (%)	159 (47.60)
Age	60.49 ± 19.36
BMI (kg/m^2^)	24.05 ± 4.52
Smoking, *n* (%)	78 (23.35)
Hypertension, *n* (%)	211 (63.17)
DM, *n* (%)	135 (40.42)
Coronary artery disease, *n* (%)	35 (10.47)
PAD, *n* (%)	2 (0.59)
Chronic HF, *n* (%)	17 (5.08)
Cerebrovascular disease, *n* (%)	43 (12.87)
BUN (mg/dL)	33.60 (15.47–69.43)
Cr (mg/dL)	1.83 (0.85–4.37)
eGFR (mL/min/1.73 m^2^)	34.35 (12.50–82.07)
CKD Stage 1 (eGFR ≥ 90)	75(22.45)
CKD stage 2 (60 ≤ eGFR ≤ 89)	53(15.87)
CKD stage 3 (30 ≤ eGFR ≤ 59)	50(14.97)
CKD stage 4 (15 ≤ eGFR ≤ 30)	53(15.87)
CKD stage 5 (eGFR < 15)	103(30.84)
Serum total protein (g/dL)	6.50 (5.80–7.10)
Serum albumin (g/dL)	3.63 (3.13–4.09)
Blood Manganese (μg/L)	8.30 (5.27–11.63)
Total cholesterol (μg/L)	157.0 (129.0–200.00)
Anemia, *n* (%)	233 (69.76)
Hb (g/dL)	11.00 (9.2–12.7)
Hct (%)	32.08 (27.25–36.70)
RDW (%)	13.40 (12.60–14.50)
Iron (μg/dL)	56.00 (37.0–77.0)
Ferritin (ng/mL)	141.08 (70.88–254.91)
TIBC (μg/dL)	241.00 (202.0–284.0)
Transferrin (mg/dL)	202.00 (164.0–236.0)
TSAT (%)	24.0 (15.50–33.30)
Erythropoietin stimulating agent, *n* (%)	44 (13.17)
Iron supplement, *n* (%)	33 (9.88)

Data are presented as mean ± standard deviation, median (interquartile range), or number (%). Abbreviations: CKD: chronic kidney disease; BMI: body mass index; DM: diabetes mellitus; PAD: peripheral artery disease; HF: heart failure; BUN: blood urea nitrogen; Cr: creatinine; eGFR: estimated glomerular filtration rate; Hb: hemoglobin; Hct: hematocrit; RDW: red blood cell distribution width; TIBC: total iron binding capacity; TSAT: transferrin saturation.

**Table 2:** Determinants of blood Mn level.

Linear Regression Model	Unadjusted		Adjusted Multivariate	
	β	*p* Value	β	*p* Value
Gender, female	0.854	0.098	1.004	0.072
Age, per 1 year	−0.049	<0.001	0.017	0.326
BMI, per 1 kg/m^2^	0.002	0.975	−0.067	0.229
Smoking	−1.588	0.009	2.069	0.001
Hypertension	−1.470	0.006	0.758	0.230
DM	0.925	0.078	−1.453	0.012
Anemia	−3.569	<0.001		
Hb, per 1 g/dL increase	0.878	<0.001	0.847	<0.001
Hct, per 1% increase	0.301	<0.001		
BUN, per 1 mg/dL increase	−0.044	<0.001		
sCr, per 1 mg/dL increase	−0.580	<0.001		
eGFR, per 1 mL/min/1.73 m^2^ increase	0.036	<0.001	0.002	0.768
Total protein, per 1 g/dL increase	1.226	<0.001		
Serum albumin, per 1 g/dL increase	1.930	<0.001	−0.041	0.935
Serum iron, per 1 μg/dL increase	0.001	0.880		
TIBC, per 1 μg/dL increase	0.025	<0.001	0.016	0.004
Transferrin, per 1 mg/dL increase	0.029	<0.001		
Serum ferritin, per 1 ng/mL increase	−0.001	0.210		
Transferrin saturation, per 1% increase	−0.035	0.024	−0.010	0.513
Erythropoietin stimulating agent	−2.565	0.001	−0.740	0.342
Iron supplement	−2.099	0.015	−0.450	0.592

**Table 3:** Poisson regression analysis with robust variance between anemia and blood Mn, clinical and nutritional factors.

Variables	Univariate			Multivariate		
	PR	*p* Value	95% CI	PR	*p* Value	95% CI
Gender, Female	1.05	0.488	0.825–1.096	1.095	0.134	0.811–1.028
Age per 1 year	1.015	<0.001	1.011–1.020	1.005	0.033	1.000–1.009
Smoking	0.897	0.244	0.747–1.077	0.922	0.305	0.789–1.077
Hypertension	1.573	<0.001	1.308–1.892	0.981	0.807	0.838–1.147
DM	1.539	<0.001	1.344–1.761	1.148	0.032	1.012–1.303
Blood Mn per 1 μg/L increase	0.947	<0.001	0.929–0.965	0.974	0.005	0.957–0.992
Ferritin 100–500 ng/mL						
≤100 ng/mL	0.763	0.003	0.637–0.914	1.008	0.919	0.869–1.169
>500 ng/mL	1.107	0.248	0.932–1.315	1.037	0.645	0.889–1.210
Transferrin 1 mg/dL increase	0.997	<0.001	0.996–0.998	1.002	0.005	1.001–1.004
Albumin per 1 μg/L increase	0.991	<0.001	0.988–0.993	0.756	<0.001	0.675–0.847
eGFR per 1 mL/min/1.73 m^2^ increase	0.971	<0.001	0.964–0.978	0.994	<0.001	0.991–0.996
Total cholesterol per 1 μg/L increase	0.997	<0.001	0.996–0.999	0.998	0.006	0.997–1.000
Use of ESA	1.492	0.001	1.357–1.640	1.033	0.561	0.866–1.082

Abbreviations: PR: prevalence ratio; CI: confidence interval; ESA; erythropoietin stimulating agent.

**Table 4:** Poisson regression analysis with robust variance for anemia in subgroups according to renal function.

Models	eGFR < 60 mL/min/m^2^		eGFR ≥ 60 mL/min/m^2^	
	PR	95% CI	PR	95% CI
Blood Mn per 1 μg/L increase				
Crude	0.965 *	0.948–0.983	0.973	0.926–1.023
Model 1	0.966 *	0. 949–0.982	0.987	0.938–1.039
Model 2	0.965 *	0.949–0.982	0.983	0.934–1.034
Model 3	0.967 *	0. 950–0.987	0.982	0.928–1.038
Model 4	0. 968 *	0.951–0.985	0.971	0. 915–1.030

Model 1: adjusting for age and sex; Model 2: adjusting for age, sex, smoking, and hypertension; Model 3: adjusting for age, sex, smoking, hypertension, ferritin 3 groups, and use of ESA; Model 4: adjusting for age, sex, smoking, hypertension, ferritin 3 groups, use of ESA, serum albumin, total cholesterol and transferrin. * *p* < 0.05.

**Table 5:** Poisson regression analysis with robust variance for anemia in subgroups according to DM.

Models	DM		Non-DM	
	PR	95% CI	PR	95% CI
Blood Mn per 1 μg/L increase				
Crude	0.975 *	0.957–0.994	0.927 *	0.897–0.958
Model 1	0.975 *	0.956–0.994	0.940 *	0.911–0.970
Model 2	0.975 *	0.956–0.995	0.941 *	0.912–0.971
Model 3	0.974 *	0.954–0.994	0.952 *	0.921–0.984
Model 4	0.982	0.964–1.001	0.949 *	0.918–0.981

Model 1: adjusting for age and sex; Model 2: adjusting for age, sex, smoking, and hypertension; Model 3: adjusting for age, sex, smoking, hypertension, ferritin 3 groups, and use of ESA; Model 4: adjusting for age, sex, smoking, hypertension, ferritin 3 groups, use of ESA, serum albumin, total cholesterol and transferrin. * *p* < 0.05.
